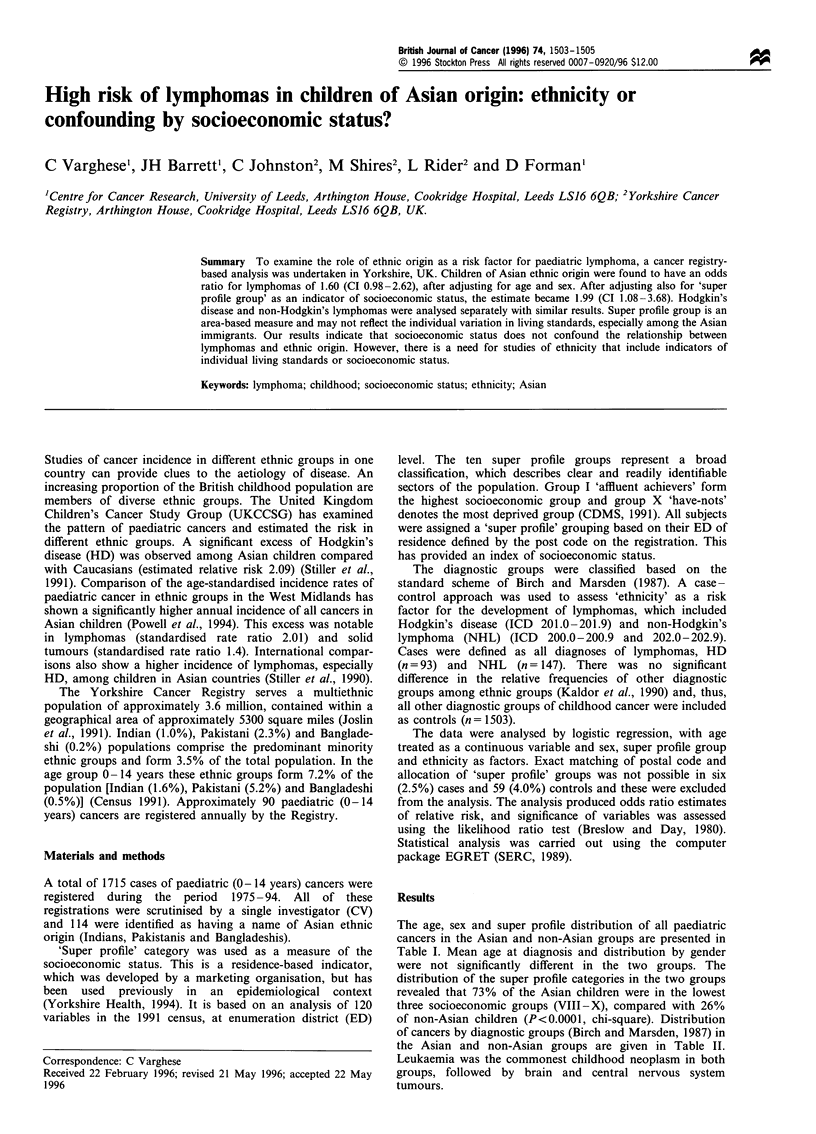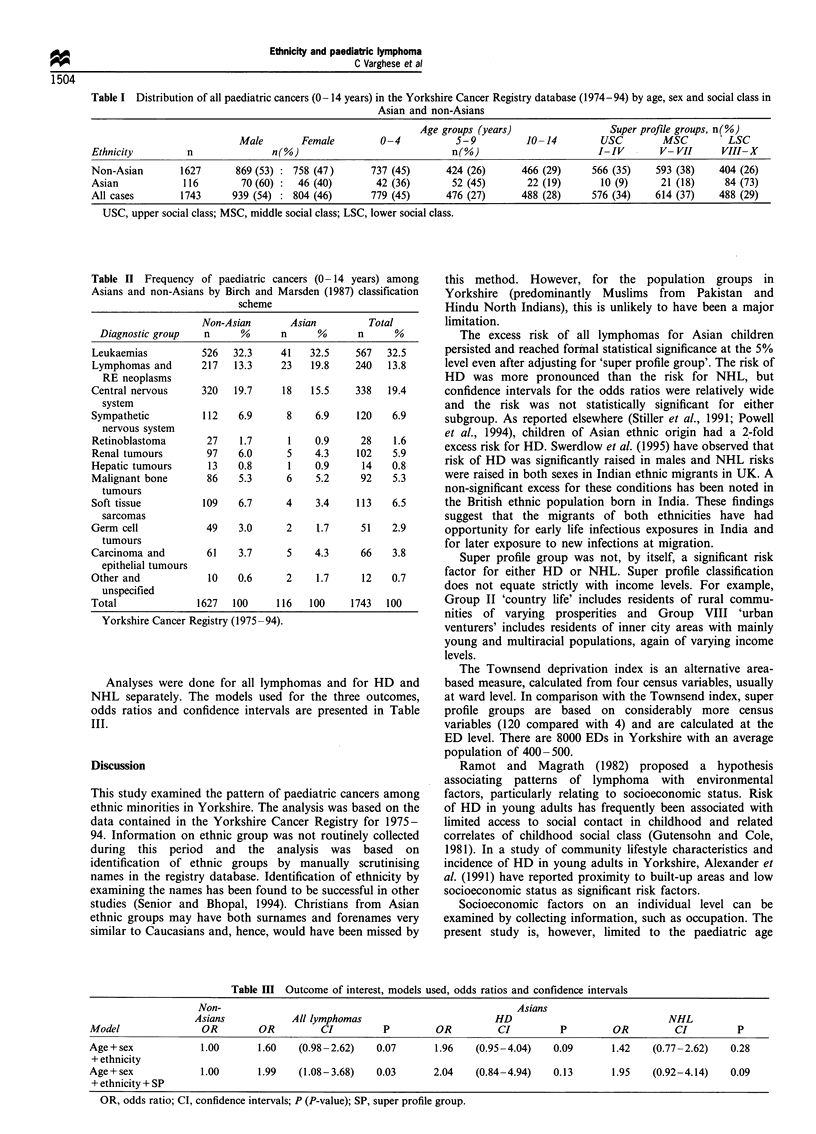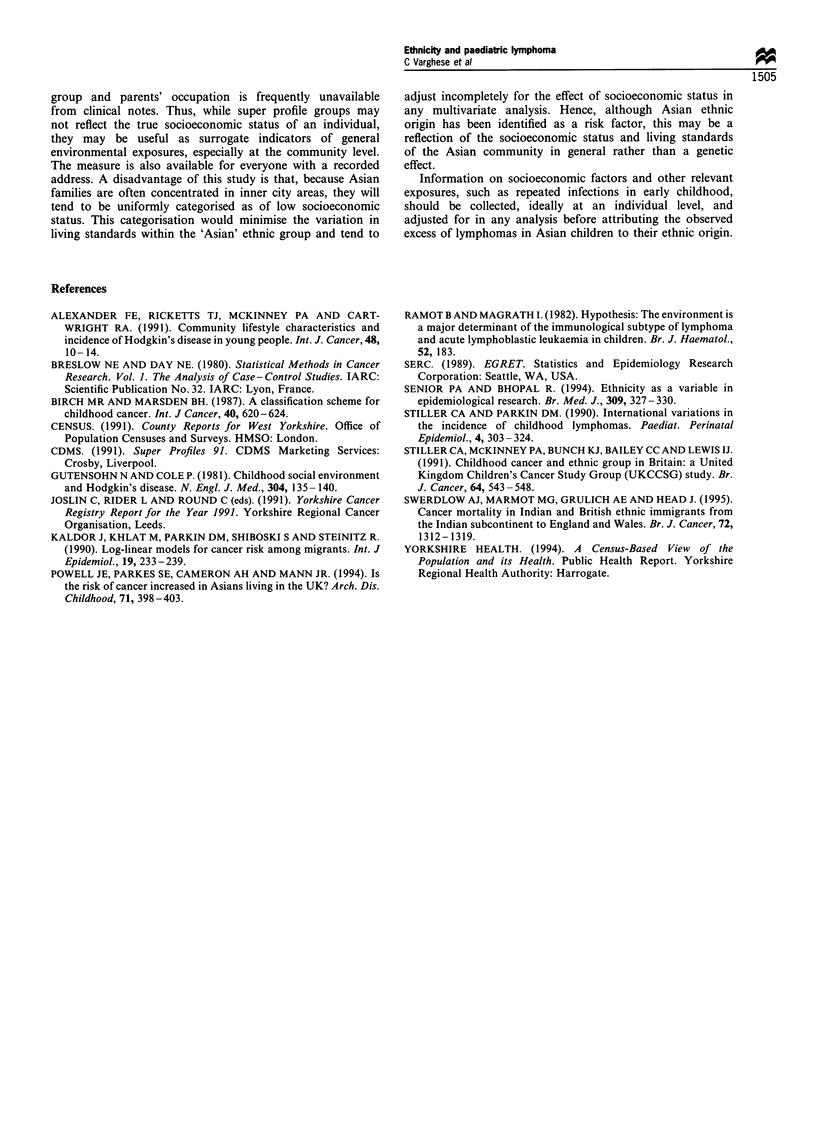# High risk of lymphomas in children of Asian origin: ethnicity or confounding by socioeconomic status?

**DOI:** 10.1038/bjc.1996.573

**Published:** 1996-11

**Authors:** C. Varghese, J. H. Barrett, C. Johnston, M. Shires, L. Rider, D. Forman

**Affiliations:** Centre for Cancer Research, University of Leeds, UK.

## Abstract

To examine the role of ethnic origin as a risk factor for paediatric lymphoma, a cancer registry-based analysis was undertaken in Yorkshire, UK. Children of Asian ethnic origin were found to have an odds ratio for lymphomas of 1.60 (CI 0.98-2.62), after adjusting for age and sex. After adjusting also for 'super profile group' as an indicator of socioeconomic status, the estimate became 1.99 (CI 1.08-3.68). Hodgkin's disease and non-Hodgkin's lymphomas were analysed separately with similar results. Super profile group is an area-based measure and may not reflect the individual variation in living standards, especially among the Asian immigrants. Our results indicate that socioeconomic status does not confound the relationship between lymphomas and ethnic origin. However, there is a need for studies of ethnicity that include indicators of individual living standards or socioeconomic status.


British Journal of Cancer (1996) 74, 1503-1505
© 1996 Stockton Press All rights reserved 0007-0920/96 $12.00

### High risk of lymphomas in children of Asian origin: ethnicity or confounding by socioeconomic status?

C Varghese[1], JH Barrett[1], C Johnston[2], M Shires[2], L Rider[2] and D Forman[1]

[1]*Centre for Cancer Research, University of Leeds, Arthington House, Cookridge Hospital, Leeds LS16 6QB;* [2]*Yorkshire Cancer Registry, Arthington House, Cookridge Hospital, Leeds LS16 6QB, UK.*

**Summary** To examine the role of ethnic origin as a risk factor for paediatric lymphoma, a cancer registry-based analysis was undertaken in Yorkshire, UK. Children of Asian ethnic origin were found to have an odds ratio for lymphomas of 1.60 (CI 0.98-2.62), after adjusting for age and sex. After adjusting also for 'super profile group' as an indicator of socioeconomic status, the estimate became 1.99 (CI 1.08-3.68). Hodgkin's disease and non-Hodgkin's lymphomas were analysed separately with similar results. Super profile group is an area-based measure and may not reflect the individual variation in living standards, especially among the Asian immigrants. Our results indicate that socioeconomic status does not confound the relationship between lymphomas and ethnic origin. However, there is a need for studies of ethnicity that include indicators of individual living standards or socioeconomic status.

**Keywords:** lymphoma; childhood; socioeconomic status; ethnicity; Asian

Studies of cancer incidence in different ethnic groups in one country can provide clues to the aetiology of disease. An increasing proportion of the British childhood population are members of diverse ethnic groups. The United Kingdom Children's Cancer Study Group (UKCCSG) has examined the pattern of paediatric cancers and estimated the risk in different ethnic groups. A significant excess of Hodgkin's disease (HD) was observed among Asian children compared with Caucasians (estimated relative risk 2.09) (Stiller *et al.*, 1991). Comparison of the age-standardised incidence rates of paediatric cancer in ethnic groups in the West Midlands has shown a significantly higher annual incidence of all cancers in Asian children (Powell *et al.*, 1994). This excess was notable in lymphomas (standardised rate ratio 2.01) and solid tumours (standardised rate ratio 1.4). International comparisons also show a higher incidence of lymphomas, especially HD, among children in Asian countries (Stiller *et al.*, 1990).

The Yorkshire Cancer Registry serves a multiethnic population of approximately 3.6 million, contained within a geographical area of approximately 5300 square miles (Joslin *et al.*, 1991). Indian (1.0%), Pakistani (2.3%) and Bangladeshi (0.2%) populations comprise the predominant minority ethnic groups and form 3.5% of the total population. In the age group 0-14 years these ethnic groups form 7.2% of the population [Indian (1.6%), Pakistani (5.2%) and Bangladeshi (0.5%)] (Census 1991). Approximately 90 paediatric (0-14 years) cancers are registered annually by the Registry.

### Materials and methods

A total of 1715 cases of paediatric (0-14 years) cancers were registered during the period 1975-94. All of these registrations were scrutinised by a single investigator (CV) and 114 were identified as having a name of Asian ethnic origin (Indians, Pakistanis and Bangladeshis).

'Super profile' category was used as a measure of the socioeconomic status. This is a residence-based indicator, which was developed by a marketing organisation, but has been used previously in an epidemiological context (Yorkshire Health, 1994). It is based on an analysis of 120 variables in the 1991 census, at enumeration district (ED) level. The ten super profile groups represent a broad classification, which describes clear and readily identifiable sectors of the population. Group I 'affluent achievers' form the highest socioeconomic group and group X 'have-nots' denotes the most deprived group (CDMS, 1991). All subjects were assigned a 'super profile' grouping based on their ED of residence defined by the post code on the registration. This has provided an index of socioeconomic status.

The diagnostic groups were classified based on the standard scheme of Birch and Marsden (1987). A case-control approach was used to assess 'ethnicity' as a risk factor for the development of lymphomas, which included Hodgkin's disease (ICD 201.0-201.9) and non-Hodgkin's lymphoma (NHL) (ICD 200.0-200.9 and 202.0-202.9). Cases were defined as all diagnoses of lymphomas, HD ($n=93$) and NHL ($n=147$). There was no significant difference in the relative frequencies of other diagnostic groups among ethnic groups (Kaldor *et al.*, 1990) and, thus, all other diagnostic groups of childhood cancer were included as controls ($n=1503$).

The data were analysed by logistic regression, with age treated as a continuous variable and sex, super profile group and ethnicity as factors. Exact matching of postal code and allocation of 'super profile' groups was not possible in six (2.5%) cases and 59 (4.0%) controls and these were excluded from the analysis. The analysis produced odds ratio estimates of relative risk, and significance of variables was assessed using the likelihood ratio test (Breslow and Day, 1980). Statistical analysis was carried out using the computer package EGRET (SERC, 1989).

### Results

The age, sex and super profile distribution of all paediatric cancers in the Asian and non-Asian groups are presented in Table I. Mean age at diagnosis and distribution by gender were not significantly different in the two groups. The distribution of the super profile categories in the two groups revealed that 73% of the Asian children were in the lowest three socioeconomic groups (VIII-X), compared with 26% of non-Asian children ($P<0.0001$, chi-square). Distribution of cancers by diagnostic groups (Birch and Marsden, 1987) in the Asian and non-Asian groups are given in Table II. Leukaemia was the commonest childhood neoplasm in both groups, followed by brain and central nervous system tumours.

Correspondence: C Varghese
Received 22 February 1996; revised 21 May 1996; accepted 22 May 1996

**Table I** Distribution of all paediatric cancers (0–14 years) in the Yorkshire Cancer Registry database (1974–94) by age, sex and social class in Asian and non-Asians

| *Ethnicity* | n | *Male* n(%) | *Female* n(%) | *0–4* | *Age groups (years)* *5–9* n(%) | *10–14* | *Super profile groups,* n(%) *USC I–IV* | *MSC V–VII* | *LSC VIII–X* |
|---|---|---|---|---|---|---|---|---|---|
| Non-Asian | 1627 | 869 (53) : | 758 (47) | 737 (45) | 424 (26) | 466 (29) | 566 (35) | 593 (38) | 404 (26) |
| Asian | 116 | 70 (60) : | 46 (40) | 42 (36) | 52 (45) | 22 (19) | 10 (9) | 21 (18) | 84 (73) |
| All cases | 1743 | 939 (54) : | 804 (46) | 779 (45) | 476 (27) | 488 (28) | 576 (34) | 614 (37) | 488 (29) |

USC, upper social class; MSC, middle social class; LSC, lower social class.

**Table II** Frequency of paediatric cancers (0–14 years) among Asians and non-Asians by Birch and Marsden (1987) classification scheme

| *Diagnostic group* | *Non-Asian* n | % | *Asian* n | % | *Total* n | % |
|---|---|---|---|---|---|---|
| Leukaemias | 526 | 32.3 | 41 | 32.5 | 567 | 32.5 |
| Lymphomas and RE neoplasms | 217 | 13.3 | 23 | 19.8 | 240 | 13.8 |
| Central nervous system | 320 | 19.7 | 18 | 15.5 | 338 | 19.4 |
| Sympathetic nervous system | 112 | 6.9 | 8 | 6.9 | 120 | 6.9 |
| Retinoblastoma | 27 | 1.7 | 1 | 0.9 | 28 | 1.6 |
| Renal tumours | 97 | 6.0 | 5 | 4.3 | 102 | 5.9 |
| Hepatic tumours | 13 | 0.8 | 1 | 0.9 | 14 | 0.8 |
| Malignant bone tumours | 86 | 5.3 | 6 | 5.2 | 92 | 5.3 |
| Soft tissue sarcomas | 109 | 6.7 | 4 | 3.4 | 113 | 6.5 |
| Germ cell tumours | 49 | 3.0 | 2 | 1.7 | 51 | 2.9 |
| Carcinoma and epithelial tumours | 61 | 3.7 | 5 | 4.3 | 66 | 3.8 |
| Other and unspecified | 10 | 0.6 | 2 | 1.7 | 12 | 0.7 |
| Total | 1627 | 100 | 116 | 100 | 1743 | 100 |

Yorkshire Cancer Registry (1975–94).

Analyses were done for all lymphomas and for HD and NHL separately. The models used for the three outcomes, odds ratios and confidence intervals are presented in Table III.

### Discussion

This study examined the pattern of paediatric cancers among ethnic minorities in Yorkshire. The analysis was based on the data contained in the Yorkshire Cancer Registry for 1975–94. Information on ethnic group was not routinely collected during this period and the analysis was based on identification of ethnic groups by manually scrutinising names in the registry database. Identification of ethnicity by examining the names has been found to be successful in other studies (Senior and Bhopal, 1994). Christians from Asian ethnic groups may have both surnames and forenames very similar to Caucasians and, hence, would have been missed by this method. However, for the population groups in Yorkshire (predominantly Muslims from Pakistan and Hindu North Indians), this is unlikely to have been a major limitation.

The excess risk of all lymphomas for Asian children persisted and reached formal statistical significance at the 5% level even after adjusting for 'super profile group'. The risk of HD was more pronounced than the risk for NHL, but confidence intervals for the odds ratios were relatively wide and the risk was not statistically significant for either subgroup. As reported elsewhere (Stiller *et al.*, 1991; Powell *et al.*, 1994), children of Asian ethnic origin had a 2-fold excess risk for HD. Swerdlow *et al.* (1995) have observed that risk of HD was significantly raised in males and NHL risks were raised in both sexes in Indian ethnic migrants in UK. A non-significant excess for these conditions has been noted in the British ethnic population born in India. These findings suggest that the migrants of both ethnicities have had opportunity for early life infectious exposures in India and for later exposure to new infections at migration.

Super profile group was not, by itself, a significant risk factor for either HD or NHL. Super profile classification does not equate strictly with income levels. For example, Group II 'country life' includes residents of rural communities of varying prosperities and Group VIII 'urban venturers' includes residents of inner city areas with mainly young and multiracial populations, again of varying income levels.

The Townsend deprivation index is an alternative area-based measure, calculated from four census variables, usually at ward level. In comparison with the Townsend index, super profile groups are based on considerably more census variables (120 compared with 4) and are calculated at the ED level. There are 8000 EDs in Yorkshire with an average population of 400–500.

Ramot and Magrath (1982) proposed a hypothesis associating patterns of lymphoma with environmental factors, particularly relating to socioeconomic status. Risk of HD in young adults has frequently been associated with limited access to social contact in childhood and related correlates of childhood social class (Gutensohn and Cole, 1981). In a study of community lifestyle characteristics and incidence of HD in young adults in Yorkshire, Alexander *et al.* (1991) have reported proximity to built-up areas and low socioeconomic status as significant risk factors.

Socioeconomic factors on an individual level can be examined by collecting information, such as occupation. The present study is, however, limited to the paediatric age

**Table III** Outcome of interest, models used, odds ratios and confidence intervals

| *Model* | *Non-Asians* *OR* | *Asians* *All lymphomas* *OR* | *CI* | P | *HD* *OR* | *CI* | P | *NHL* *OR* | *CI* | P |
|---|---|---|---|---|---|---|---|---|---|---|
| Age + sex + ethnicity | 1.00 | 1.60 | (0.98–2.62) | 0.07 | 1.96 | (0.95–4.04) | 0.09 | 1.42 | (0.77–2.62) | 0.28 |
| Age + sex + ethnicity + SP | 1.00 | 1.99 | (1.08–3.68) | 0.03 | 2.04 | (0.84–4.94) | 0.13 | 1.95 | (0.92–4.14) | 0.09 |

OR, odds ratio; CI, confidence intervals; *P* (*P*-value); SP, super profile group.

group and parents' occupation is frequently unavailable from clinical notes. Thus, while super profile groups may not reflect the true socioeconomic status of an individual, they may be useful as surrogate indicators of general environmental exposures, especially at the community level. The measure is also available for everyone with a recorded address. A disadvantage of this study is that, because Asian families are often concentrated in inner city areas, they will tend to be uniformly categorised as of low socioeconomic status. This categorisation would minimise the variation in living standards within the 'Asian' ethnic group and tend to adjust incompletely for the effect of socioeconomic status in any multivariate analysis. Hence, although Asian ethnic origin has been identified as a risk factor, this may be a reflection of the socioeconomic status and living standards of the Asian community in general rather than a genetic effect.

Information on socioeconomic factors and other relevant exposures, such as repeated infections in early childhood, should be collected, ideally at an individual level, and adjusted for in any analysis before attributing the observed excess of lymphomas in Asian children to their ethnic origin.

### References

ALEXANDER FE, RICKETTS TJ, MCKINNEY PA AND CARTWRIGHT RA. (1991). Community lifestyle characteristics and incidence of Hodgkin's disease in young people. *Int. J. Cancer*, **48**, 10–14.

BRESLOW NE AND DAY NE. (1980). *Statistical Methods in Cancer Research. Vol. 1. The Analysis of Case–Control Studies*. IARC: Scientific Publication No. 32. IARC: Lyon, France.

BIRCH MR AND MARSDEN BH. (1987). A classification scheme for childhood cancer. *Int. J Cancer*, **40**, 620–624.

CENSUS. (1991). *County Reports for West Yorkshire*. Office of Population Censuses and Surveys. HMSO: London.

CDMS. (1991). *Super Profiles 91*. CDMS Marketing Services: Crosby, Liverpool.

GUTENSOHN N AND COLE P. (1981). Childhood social environment and Hodgkin's disease. *N. Engl. J. Med.*, **304**, 135–140.

JOSLIN C, RIDER L AND ROUND C (eds). (1991). *Yorkshire Cancer Registry Report for the Year 1991*. Yorkshire Regional Cancer Organisation, Leeds.

KALDOR J, KHLAT M, PARKIN DM, SHIBOSKI S AND STEINITZ R. (1990). Log-linear models for cancer risk among migrants. *Int. J Epidemiol.*, **19**, 233–239.

POWELL JE, PARKES SE, CAMERON AH AND MANN JR. (1994). Is the risk of cancer increased in Asians living in the UK? *Arch. Dis. Childhood*, **71**, 398–403.

RAMOT B AND MAGRATH I. (1982). Hypothesis: The environment is a major determinant of the immunological subtype of lymphoma and acute lymphoblastic leukaemia in children. *Br. J. Haematol.*, **52**, 183.

SERC. (1989). *EGRET*. Statistics and Epidemiology Research Corporation: Seattle, WA, USA.

SENIOR PA AND BHOPAL R. (1994). Ethnicity as a variable in epidemiological research. *Br. Med. J.*, **309**, 327–330.

STILLER CA AND PARKIN DM. (1990). International variations in the incidence of childhood lymphomas. *Paediat. Perinatal Epidemiol.*, **4**, 303–324.

STILLER CA, MCKINNEY PA, BUNCH KJ, BAILEY CC AND LEWIS IJ. (1991). Childhood cancer and ethnic group in Britain: a United Kingdom Children's Cancer Study Group (UKCCSG) study. *Br. J. Cancer*, **64**, 543–548.

SWERDLOW AJ, MARMOT MG, GRULICH AE AND HEAD J. (1995). Cancer mortality in Indian and British ethnic immigrants from the Indian subcontinent to England and Wales. *Br. J. Cancer*, **72**, 1312–1319.

YORKSHIRE HEALTH. (1994). *A Census-Based View of the Population and its Health*. Public Health Report. Yorkshire Regional Health Authority: Harrogate.